# Crovirin, a Snake Venom Cysteine-Rich Secretory Protein (CRISP) with Promising Activity against Trypanosomes and *Leishmania*

**DOI:** 10.1371/journal.pntd.0003252

**Published:** 2014-10-16

**Authors:** Camila M. Adade, Ana Lúcia O. Carvalho, Marcelo A. Tomaz, Tatiana F. R. Costa, Joseane L. Godinho, Paulo A. Melo, Ana Paula C. A. Lima, Juliany C. F. Rodrigues, Russolina B. Zingali, Thaïs Souto-Padrón

**Affiliations:** 1 Instituto de Microbiologia Paulo de Góes, Universidade Federal do Rio de Janeiro, Rio de Janeiro, Brazil; 2 Instituto Nacional de Ciência e Tecnologia de Biologia Estrutural e Bioimagem, Rio de Janeiro, Brazil; 3 Instituto de Biquímica Médica, Universidade Federal do Rio de Janeiro, Rio de Janeiro, Brazil; 4 Instituto de Ciências Biomédicas, Universidade Federal do Rio de Janeiro, Rio de Janeiro, Brazil; 5 Instituto de Biofísica Carlos Chagas Filho, Universidade Federal do Rio de Janeiro, Rio de Janeiro, Brazil; 6 Instituto Nacional de Metrologia, Qualidade e Tecnologia, Inmetro, Rio de Janeiro, Brazil; 7 Núcleo Multidisciplinar de Pesquisa em Biologia (NUMPEX-BIO), Polo Avançado de Xerém, Universidade Federal do Rio de Janeiro, Duque de Caxias, Brazil; Northeastern University, United States of America

## Abstract

**Background:**

The neglected human diseases caused by trypanosomatids are currently treated with toxic therapy with limited efficacy. In search for novel anti-trypanosomatid agents, we showed previously that the *Crotalus viridis viridis* (Cvv) snake venom was active against infective forms of *Trypanosoma cruzi*. Here, we describe the purification of crovirin, a cysteine-rich secretory protein (CRISP) from Cvv venom with promising activity against trypanosomes and *Leishmania*.

**Methodology/Principal Findings:**

Crude venom extract was loaded onto a reverse phase analytical (C8) column using a high performance liquid chromatographer. A linear gradient of water/acetonitrile with 0.1% trifluoroacetic acid was used. The peak containing the isolated protein (confirmed by SDS-PAGE and mass spectrometry) was collected and its protein content was measured. *T. cruzi* trypomastigotes and amastigotes, *L. amazonensis* promastigotes and amastigotes and *T. brucei rhodesiense* procyclic and bloodstream trypomastigotes were challenged with crovirin, whose toxicity was tested against LLC-MK_2_ cells, peritoneal macrophages and isolated murine *extensor digitorum longus* muscle. We purified a single protein from Cvv venom corresponding, according to Nano-LC MS/MS sequencing, to a CRISP of 24,893.64 Da, henceforth referred to as crovirin. Human infective trypanosomatid forms, including intracellular amastigotes, were sensitive to crovirin, with low IC_50_ or LD_50_ values (1.10–2.38 µg/ml). A considerably higher concentration (20 µg/ml) of crovirin was required to elicit only limited toxicity on mammalian cells.

**Conclusions:**

This is the first report of CRISP anti-protozoal activity, and suggests that other members of this family might have potential as drugs or drug leads for the development of novel agents against trypanosomatid-borne neglected diseases.

## Author Summary

The pathogenic trypanosomatid parasites of the genera *Leishmania* and *Trypanosoma* infect over 20 million people worldwide, with an annual incidence of ∼3 million new infections. An additional 400 million people are at risk of infection by exposure to parasite-infected insects which act as disease vectors. Trypanosomatid-borne diseases predominant in poorer nation and are considered neglected, having failed to attract the attention of the pharmaceutical industry. However, novel therapy is sorely needed for *Trypanosoma* and *Leishmania* infections, currently treated with ‘dated’ drugs that are often difficult to administer in resource-limiting conditions, have high toxicity and are by no means always successful, partly due to the emergence of drug resistance. The last few decades have witnessed a growing interest in examining the potential of bioactive toxins and poisons as drugs or drug leads, as well as for diagnostic applications. In this context, we isolated and purified crovirin, a protein from the *Crotalus viridis viridis* (Cvv) snake venom capable to inhibiting and/or lysing infective forms of trypanosomatid parasites, at concentrations that are not toxic to host cells. This feature makes crovirin a promising candidate protein for the development of novel therapy against neglected diseases caused by trypanosomatid pathogens.

## Introduction

The pathogenic trypanosomatids from the genera *Leishmania* and *Trypanosoma* infect over 20 million people worldwide, with an annual incidence of ∼3 million new infections in at least 88 countries. An additional 400 million people are at risk of infection by exposure to insect vectors harboring parasites [1]–[3]. *Leishmania* and trypanosome infections predominate in poorer nations, and are considered neglected diseases that have “fallen below the radar of modern drug discovery” [4].

*Leishmania* parasites cause five different disease forms – cutaneous (CL), mucocutaneous (MCL), diffuse cutaneous leishmaniasis (DCL), post-kala-azar dermal leishmaniasis (PKDL) and visceral leishmaniasis (VL, also known as ‘black fever’ or ‘kala-azar’ in India) [5]. VL is the most severe and debilitating form of leishmaniasis, and can be fatal if left untreated. First-line treatment for leishmaniasis is based on pentavalent antimonials such as meglumine antimoniate (Glucantime) and sodium stibogluconate (Pentostan). Amphotericin B and pentamidine are used as second-line drugs in patients resistant to first-line therapy [1], [6]. Recently, miltefosine has been used in India as part of combination therapy regimens to treat VL, and the largest increase in miltefosine activity was seen in combination with amphotericin B [7], [8].

There are two forms of HAT (also known as sleeping sickness), caused by two subspecies of *T. brucei* parasites (*T. b. gambiense* or *T. b. rhodesiense*). Both HAT forms culminate in parasite invasion of the central nervous system, with gradual nervous system damage if untreated. The currently used anti-HAT drugs - melarsoprol, eflornithine, pentamidine, and suramin - are highly toxic and have lost efficacy in several regions. Also, treatment is difficult to administer in resource-limiting conditions, and often unsuccessful [9], [10].

Chagas' disease, caused by *T. cruzi*, affects the cardiovascular, gastrointestinal, and nervous systems of human hosts and has become, in recent decades, a worldwide public health problem due to travelers and migratory flow [2], [11]. Chagas' disease chemotherapy is based on the use of nifurtimox and benznidazole, two very toxic nitroheterocyclic compounds with modest efficacy (especially against late stage chronic disease), and ‘plagued’ by the emergence of drug resistance [12].

Given the high toxicity and limited efficacy of current treatments for leishmaniasis, Chagas' disease and HAT, the development of novel chemotherapeutics against these neglected diseases is essential. Animal venoms and poisons are natural libraries of bioactive compounds with potential to yield novel drugs or drug leads for pharmacotherapeutics [13]. In particular, snake venoms have proven to be interesting sources of potential novel agents against neglected diseases, including Chagas' disease [14]– and leishmaniasis [18]–[23].

Cysteine-rich secretory proteins (CRISPs) are single chain bioactive polypeptides with molecular masses of ∼20–30 kDa found in snake venom, reptilian venom ducts [24]–[26] and also in the salivary glands, pancreatic tissues, reproductive tracts [27]–[31]. In mammals, CRISPs are also expressed at low levels in non-reproductive tissues and organs, including skeletal muscle, spleen and thymus [32]. CRISPs belong to the CAP (Crisp, antigen 5, and pathogenesis-related) superfamily of proteins [33].

CRISP amino acid sequences have high degree of sequence identity and similarity, and include a highly conserved pattern of 16 cysteine residues which form 8 disulfide bonds [34]. Ten of these cysteine residues form an integral part of a well-conserved cysteine-rich domain at the C-terminus, although CRISP N-terminal sequences are overall more conserved than other regions of these proteins [33]–[35]. Snake venom CRISPs belong to the CRISP-3 subfamily [36], one of four subgroups of CRISPs, according to amino acid sequence homology. Most biological targets of snake venom CRISPs described to date are ion channels [37]–[43], although the functions and the molecular targets of most snake venom CRISPs remain to be determined. Some snake venom CRISPs had their biological activities tested on crickets and cockroaches [35]. Snake venom CRISPs have been shown to block the activity of L-type Ca^2+^ and/or K^+^-channels and also of cyclic nucleotide-gated (CNG) ion channels, thereby preventing the contraction of smooth muscle cells [26], [37], [40]–[43]. The CRISPs catrin, piscivorin and ophanin, from the snake *Crotalus atrox*, caused moderate blockage of L-type calcium channels, partially inhibiting the contraction of smooth fibers from mouse caudal arteries [26]. The *Philodryas patagoniensis* (green snake) CRISP patagonin was capable of generating myotoxicity when injected into the gastrocnemius muscle, but did not induce edema formation, haemorrhage or inhibition on platelet aggregation [44]. Despite their myotoxicity, there are no reports of CRISP protein lethality to mice, in concentrations of up to 4.5 mg/kg [35], [45], and patagonin did not induce systemic alterations in mice, or histological changes in tissues from the cerebellum, brain, heart, liver and spleen [44].

In a previous publication, we showed that crude venom from the rattlesnake *Crotalus viridis viridis* had anti-parasitic activity against all forms of *T. cruzi*, and could be a valuable source of molecules for the development of new drugs against Chagas' disease [46]. In search for the molecular source of the anti-parasitic activity found in Cvv crude venom, we purified a Cvv CRISP that will be henceforth referred to as ‘crovirin’. Here, we describe the purification, biochemical characterization and biological activity of crovirin against pathogenic trypanosomatids parasites and mammalian cells, showing that crovirin is active against infective developmental forms of trypanosomes and *Leishmania*, at doses that elicit no or minimal toxic effects on human cells.

## Methods

### Venom samples, compounds and reagents

Crude venom from the rattlesnake *Crotalus viridis viridis* (Cvv) and adjuvants such as parasites growth media, were purchased from Sigma–Aldrich Chemical Co (St. Louis, MO, USA). Benznidazole (Bz) (Laboratório Farmacêutico do Estado de Pernambuco [LAFEPE], Brazil), diminazene aceturate (Berenil [Ber], Hoechst Veterinãr GmbH, München, Germany), and Amphotericin B (Amp-B) (Sigma) were used as a reference drugs for Chagas disease, sleeping sickness and leishmaniasis treatment, respectively. The material and reagents used in SDS-PAGE were from Bio-Rad Laboratories, Inc. Molecular weigh markers LMW were from Fermentas Life Sciences. Mass spectrometry grade Trypsin Gold was from Promega. All other reagents and chemicals were from Merck (Darmstadt, Germany), Tedia Company and Eurofarma Laboratórios SA.

### Purification of Crovirin

Lyophilized Cvv venom (10 mg) was dissolved in 1 ml of 20 mM Tris–HCl, 150 mM NaCl, pH 8.8 and centrifuged at 5,000 *g* for 2 min. The supernatant was applied onto a reverse phase analytical C_8_ column (5 µm, 250×4.6 mm) (Kromasil, Sweeden), previously equilibrated with the same buffer. Venom proteins were separated by reverse phase HPLC (Shimadzu, Japan). Fractions (0.7 ml/tube) were collected at a 1 ml/h flowrate. A linear gradient of water/acetonitrile containing 0.1% trifluoroacetic acid (TFA) was used. The elution profile was monitored by absorption at 280 nm, and the molecular homogeneity of the relevant fractions was verified by SDS-PAGE. Fractions containing protein peaks were dried in a Speed-Vac (Savant, Thermo Scientific, USA) and resuspended in distilled water prior to protein quantification by the Bradford method. Molecular mass determination was performed by MALDI-TOF and by electrospray ionization (ESI) mass spectrometry using a Voyager-DE Pro and a QTrap 2000 (both from Applied Biosystems), respectively.

### In-gel digestion

Protein bands were excised from Coomassie Brilliant Blue-stained SDS-PAGE gels and cut into smaller pieces, which were destained with 25 mM NH_4_HCO_3_ in 50% acetonitrile for 12 h. The pieces obtained from the non-reducing gels were reduced in a solution of 10 mM dithiothreitol and 25 mM NH_4_HCO_3_ for 1 h at 56°C, and then alkylated in a solution of 55 mM iodoacetamide and 25 mM NH_4_HCO_3_, for 45 min in the dark. The solution was removed, the gel pieces were washed with 25 mM NH_4_HCO_3_ in 50% acetonitrile, and then dehydrated in 100% acetonitrile. Finally, all pieces from reducing and non-reducing gels were air-dried, rehydrated in a solution of 25 mM NH_4_HCO_3_ containing 100 ng of trypsin, and digested overnight at 37°C. Tryptic peptides were then recovered in 10 µl of 0.1% TFA in 50% acetonitrile.

### Nano LC-MS/MS mass spectrometry

The peptides extracted from gel pieces were loaded into a Waters Nano Acquity system (Waters, MA, USA) and desalted on-line using a Waters Symmetry C18 180 µm×20 mm, 5 µm trap column. The typical sample injection volume was 7.5 µl, and liquid chromatography (LC) was performed by using a BEH 130 C18 100 µm×100 mm, 1.7 µm column (Waters, MA, USA) and eluting (0.5 µl/min) with a linear gradient of 10–40% acetonitrile, containing 0.1% formic acid. Electrospray tandem mass spectra were performed in a Q-Tof quadrupole/orthogonal acceleration time-of-flight spectrometer (Waters, Milford, MA) linked to a nano ACQUITY system (Waters) capillary chromatograph. The ESI voltage was set at 3300 V, the source temperature was 80°C and the cone voltage was 30 V. The instrument control and data acquisition were conducted by a MassLynx data system (Version 4.1, Waters), and experiments were performed by scanning from a mass-to-charge ratio (*m*/*z*) of 400–2000 using a scan time of 1 s, applied during the whole chromatographic process. The mass spectra corresponding to each signal from the total ion current (TIC) chromatogram were averaged, allowing for accurate molecular mass measurements. The exact mass was determined automatically using Q-Tof's LockSpray (Waters, MA, USA). Data-dependent MS/MS acquisitions were performed on precursors with charge states of 2, 3 or 4 over a range of 50–2000 *m*/*z*, and under a 2 *m*/*z* window. A maximum of three ions were selected for MS/MS from a single MS survey. Collision-induced dissociation (CID) MS/MS spectra were obtained using argon as the collision gas at a pressure of 40 psi, and the collision voltage varied between 18 and 90 V, depending on the mass and charge of the precursor. The scan rate was 1 scan/s. All data were processed using the ProteinLynx Global server (version 2.5, Waters). The processing automatically lock mass calibrated the *m*/*z* scale of both the MS and the MS/MS data utilizing a lock spray reference ion. The MS/MS data were also charge-state deconvoluted and deisotoped with the maximum entropy algorithm MaxEnt 3 (Waters, MA, USA).

### Mass spectrometry data analysis

Proteins corresponding to the tryptic peptides from peak 3 were identified by correlation of tandem mass spectra and the NCBInr database of proteins (Version 050623), using the MASCOT software (Matrix Science, version 2.1). Settings allowed for one missed cleavage per peptide, and an initial mass tolerance of 0.2 Da was used in all searches. Cysteines were assumed to be carbamidomethylated, and a variable modification of methionine (oxidation) was allowed. Identification was considered positive when at least two peptides matched the protein sequence with a mass accuracy of less than 0.2 Da.

### Parasites

*T. cruzi* tissue culture trypomastigotes (CL-Brener clone) were obtained from the supernatants of 5 to 6-day-old infected LLC-MK_2_ cells maintained in RPMI-1640 medium (Sigma) supplemented with 2% FCS for 5–6 days at 37°C in a humidified 5% CO_2_. Theses trypomastigotes were also used to obtain intracellular amastigotes in macrophage cultures.

The MHOM/BR/75/Josefa strain of *L. amazonensis*, isolated from a patient with DCL by C. A. Cuba-Cuba (Universidade de Brasilia, Brazil), was used in the present study. Amastigote forms were maintained by hamster footpad inoculation, while promastigotes were cultured axenically in Warren's medium [47] supplemented with 10% fetal bovine serum (FBS) at 25°C. Infective promastigotes were used to obtain intracellular amastigotes in macrophage cultures, as described previously [48]. Bloodstream form (BSF) *T. brucei rhodesiense* (strain IL1852) were cultivated in HMI-9 medium (Invitrogen) supplemented with 10% inactivated FBS (Biosera-South America) and 10% of serum plus supplement (SAFC Bioscience, USA), at 37°C in a humidified 5% CO_2_ incubator [49]. Procyclic-form (PCF) *T. brucei rhodesiense* (strain 457) were grown in SDM-79 medium (LGC Biotecnologia) supplemented with 10% heat-inactivated FBS, at 28°C [50].

### Ethics statement

In this study, we used 5-week-old female CF1 mice as sources of peritoneal macrophages and of muscle sample for *ex vivo* assays (described below). All animal experimentation protocols received the approval by the Commission to Evaluate the Use of Research Animals (CAUAP, from the Carlos Chagas Filho Biophysics Institute - IBCCF), and by the Ethics Committee for Animal Experimentation (Health Sciences Center, Federal University of Rio de Janeiro – UFRJ) (Protocol no. IBCCF 096/097/106), in agreement with Brazilian federal law (11.794/2008, Decreto n° 6.899/2009). We followed institutional guidelines on animal manipulation, adhering to the “Principles of Laboratory Animal Care” (National Society for Medical Research, USA) and the “Guide for the Care and Use of Laboratory Animals” (National Academy of Sciences, USA).

### Parasite cytotoxicity assays

Crovirin was purified as described above and stored at −20°C, in 3.6 mg/ml stock solutions prepared in PBS (pH 7.2). All experiments were carried out in triplicates. Stock solutions of Bz (14 mg/ml) and Amp-B (10 mg/ml) were prepared in dimethyl sulfoxide (DMSO), and the final concentration of the solvent never exceeded 0.5%, which is not toxic for parasites and mammalian cells. Ber stock solution (0.188 mg/ml) was prepared in pyrogen-free water.

Axenically grown parasite forms were treated with crovirin for up to 72 h in the same culture conditions used for growth (described above). The following crovirin concentrations were used to treat axenic forms: 1.2–4.8 µg/ml (*L. amazonensis* promastigotes) and 0.6–4.8 µg/ml crovirin (*T. brucei rhodesiense* BSF and PCF). IC_50_ values were calculated based on daily counting of formalin-fixed parasites using a hemocytometer. Positive controls were run in parallel with 4.7 µg/ml Amp-B [51] and 39.8 ng/ml Ber [52], respectively.

*T. cruzi* tissue culture trypomastigotes were treated with crovirin (0.45–4.8 µg/ml) at a density of 1×10^6^ cells/ml, for 24 h at 37°C (in RPMI media containing 10% FCS). LD_50_ (50% trypomastigote lysis) values were determined based on direct counting of formalin-fixed parasites using a hemocytometer. Bz was used as reference drug, in a 3.39 µg/ml concentration [53].

To evaluate the effects of crovirin on *T. cruzi* and *L. amazonensis* intracellular amastigotes, peritoneal macrophages from CF1 mice were harvested by washing with RPMI medium (Sigma), and plated in 24-well tissue culture chamber slides, allowing them to adhere to the slides for 24 h at 37°C in 5% CO_2_. Adherent macrophages were infected with tissue culture *T. cruzi* trypomastigotes (at 37°C) or *L. amazonensis* metacyclic promastigotes (at 35°C) at a macrophage-to-parasite ratio of 1∶10, for 2 h. After this period, non-internalized parasites were removed by washing, cultures were incubated for 24 h in RPMI with 10% FCS, and fresh medium with crovirin (0.45–3.6 µg/ml for *T. cruzi*, and 0.6–9.6 µg/ml for *L. amazonensis*) was added daily for 72 h. At different time-points (24, 48 and 72 h) cultures were fixed with 4% paraformaldehyde in PBS (pH 7.2) and stained with Giemsa for 15 min. The percentage of infected cells and the number of parasites per 100 cells were determined by light microscopy examination. Positive controls of *T. cruzi* and *L. amazonensis* amastigotes infected cells were run in parallel with cultures treated with 0.73 µg/ml Bz [53] and 0.07 µg/ml Amp-B [54], respectively.

### Mammalian cell cytotoxicity assays

LLC-MK_2_ cells were maintained in RPMI medium supplemented with 10% FCS. Prior to treatment with crovirin, cells were seeded in 24-well plates containing glass coverslips and incubated in RPMI medium supplemented with 10% FCS for 24 h at 37°C. Cells were then treated with 4.8, 10 and 20 µg/ml crovirin at 37°C for 72 h. LC_50_ values (concentrations that reduces by 50% the cellular viability) for crovirin were calculated from daily counts of the number of viable cells, using trypan blue as an exclusion dye. At least 500 cells were examined per well, on a Zeiss Axiovert light microscope (Oberkochen, Germany).

In addition, mouse peritoneal macrophages were seeded on 96-well plates, incubated in RPMI medium with 10% FCS for 24 h at 37°C and treated with 4.8, 10 and 20 µg/ml crovirin at 37°C, for 72 h. After this period, cells were washed with PBS (pH 7.2), and the wells were filled with RPMI medium without phenol red containing 10 mM glucose and 20 µl of a solution of 2 mg/ml MTS (3-(4,5-dimethylthiazol-2-yl)-5-(3-carboxymethoxyphenyl)-2-(4-sulfophenyl)-2H-tetrazolium salt) and 0.92 mg/ml PMS (phenazine methosulfate), prepared according to the manufacturer's instructions (Promega, Madison, WI, USA). Following 3 h of incubation at 37°C, formation of a soluble formazan product by viable cells was measured using a plate reader, by absorbance at 490 nm. All cytotoxicity experiments were carried out in triplicates.

### *Ex vivo* mytotoxicity assay

The myotoxicity of crovirin was studied *ex vivo* using a muscle creatin kinase (CK) activity assay [55]. The analysis consisted of monitoring the rate of CK release from isolated mouse *extensor digitorum longus* (EDL) muscle bathed in a solution containing crovirin (10 µg/ml). Adult male and female Swiss mice (25.0±5.0 g) were anesthetized with ethyl ether and killed by cervical dislocation. EDL muscles were collected, freed from fat and tendons, dried and weighed. Muscle samples were then homogenized in 2 ml saline/0.1% albumin and their CK content was determined using a commercial diagnostic kit (Bioclin, Brazil). Four EDL muscles were mounted vertically on a cylindrical chamber and superfused continuously with Ringer's solution equilibrated with 95% O_2_/5% CO_2_. At 30 to 90-min intervals, the perfusing solution was collected and replaced with fresh solution. The collected EDL samples were used for the measurement of CK activity as described above. Muscles were weighed at the end of the experiment (2 h later). Enzyme activity is reported as international units corrected for muscle mass.

### Statistical analysis

Mean value comparisons between control and treated groups were performed using the Kruskal-Wallis test in the BioEstat 2.0 program for Windows. Differences with p≤0.05 were considered statistically significant.

## Results

### Purification of crovirin, a CRISP from the snake venom of *C. viridis viridis*

**Figure 1:**
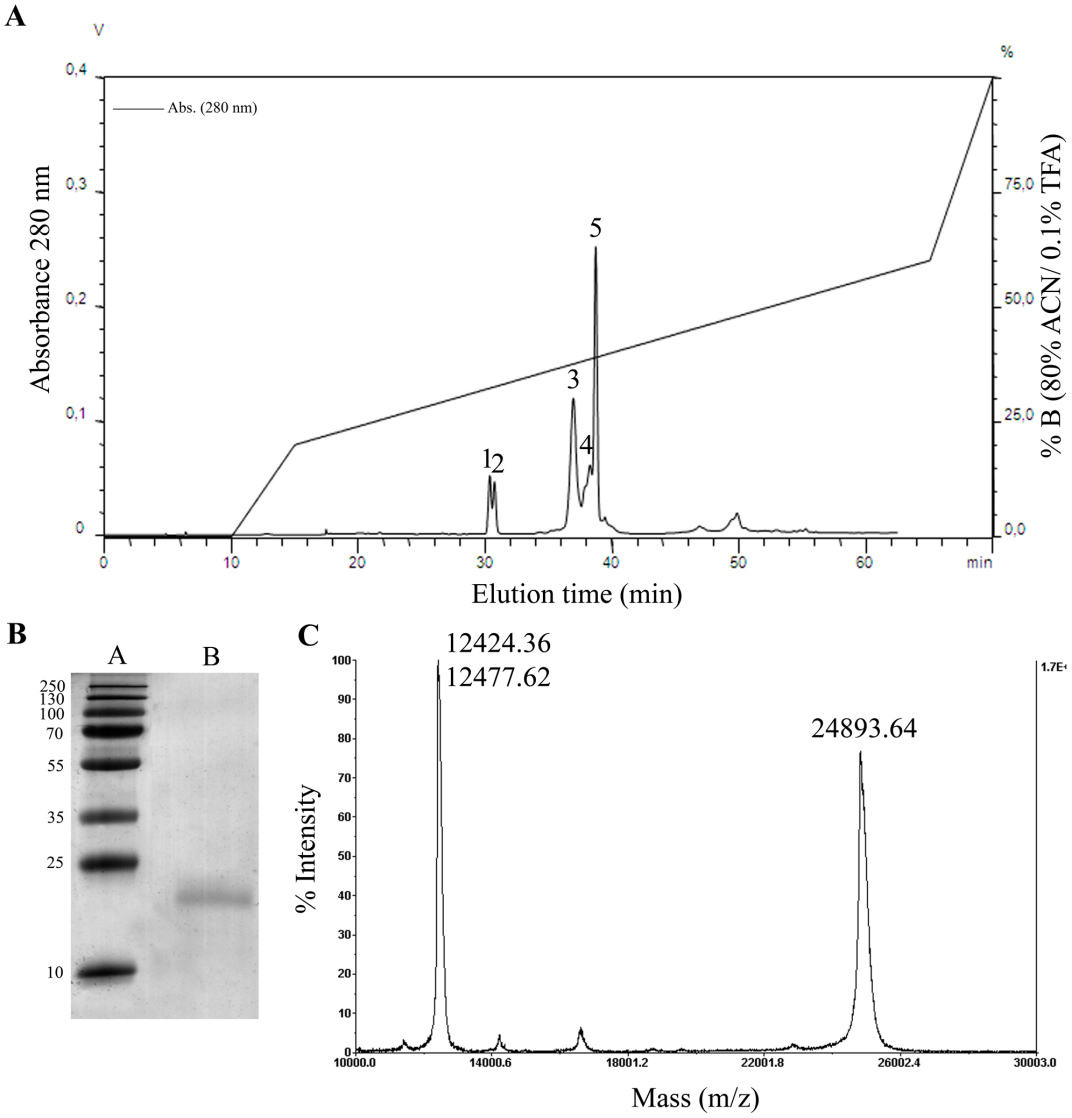
(**A**) **Crovirin purification from Cvv venom using a reverse phase analytical C_8_ column, where the protein was eluted as peak 3.** (**B**) SDS-PAGE analysis of peak 3 (lane B) containing the purified crovirin protein under reducing conditions. The gel was stained with Coomassie blue. Lane A, molecular weight markers. (**C**) MALDI-TOF mass spectrometry analyses of the intact protein yielded a molecular mass of 24,893.64 Da. The peaks of 12,424.36 and 12,477.62 Da correspond to doubly-charged (z = 2) cationic forms of crovirin.

**Figure 2:**
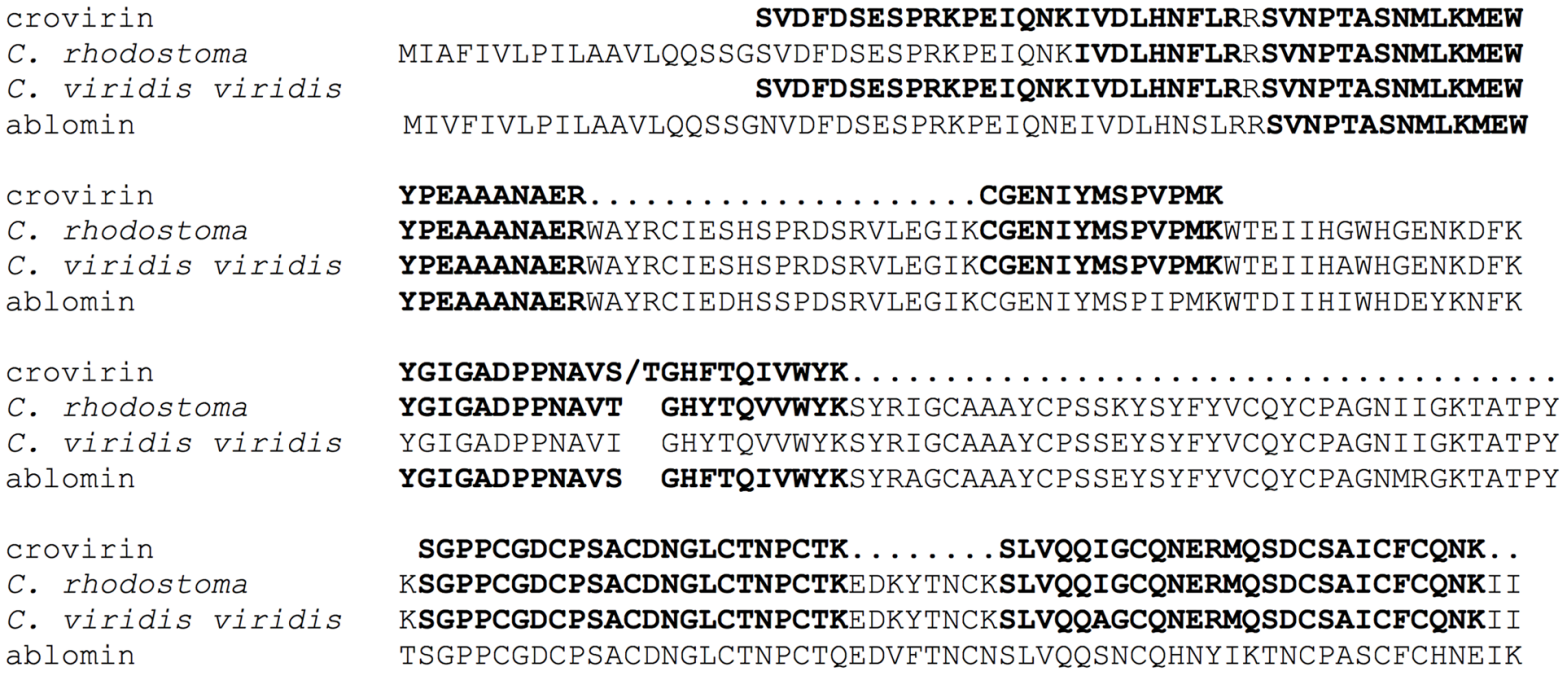
Alignment of crovirin peptide sequences (identified by MALDI-TOF-TOF) and homologous sequences from *Calloselasma rhodostoma* (gi:190195317), *Crotalus viridis viridis* (gi:190195319) and *Gloydius blomhoffi* (ablomin protein- gi:48428846) deposited in GenBank. Bold letters highlight identical residues in the same position of all protein sequences.

In a previous study, we showed that the Cvv venom had anti-parasitic activity against *T. cruzi* [46]. Preliminary analysis of Cvv venom fractions by reverse-phase chromatography (not shown) indicated that the activity eluted with fractions containing peak 3 of the chromatographic profile (**Fig. 1A**). Thus, we analyzed the main chromatographic fraction corresponding to peak 3 by SDS-PAGE and MALDI-TOF mass spectrometry (**Fig. 1B–C**). SDS-PAGE analysis of peak 3 showed a single polypeptide, with a relative molecular mass of 24 kDa (**Fig. 1B**) and 28 kDa (data not shown), under reducing and non-reducing conditions, respectively. We will refer to this protein henceforth as crovirin. MALDI-TOF analysis of the intact protein showed a molecular mass of 24,893.64 Da (**Fig. 1C**). The peaks of 12,424.36 and 12,477.62 Da in the MS profile correspond to doubly-charged (z = 2) cationic forms of the protein. The amino acid sequence of tryptic crovirin peptides (produced by Nano LC-MS/MS mass spectrometry analysis) is nearly identical to a partial sequence of a Cvv CRISP (GenBank gi:190195319) (**Fig. 2**). The MS/MS-derived sequences are also nearly identical to those of a CRISP protein from *Calloselasma rhodostoma* (GenBank gi:190195317) and have high degree of sequence similarity to several other snake venom CRISPs, including ablomin (**Fig. 2**). The MS/MS spectrum of the fragmented peptide ions was matched by MASCOT displayed a coverage of 48% of identical peptides, with a p≥355 indicating extensive homology to the CRISP from *C. rhodostoma*. The MS results strongly suggested that a CRISP from Cvv snake venom had been purified, and corresponded to crovirin.

### Crovirin has significant anti-parasitic activity against infective forms of trypanosomatids, with minimal toxicity

First of all, we investigated crovirin citotoxicity over mammalian host cells before proceeding with our analysis of the anti-parasitic activity of this venom protein.

**Figure 3:**
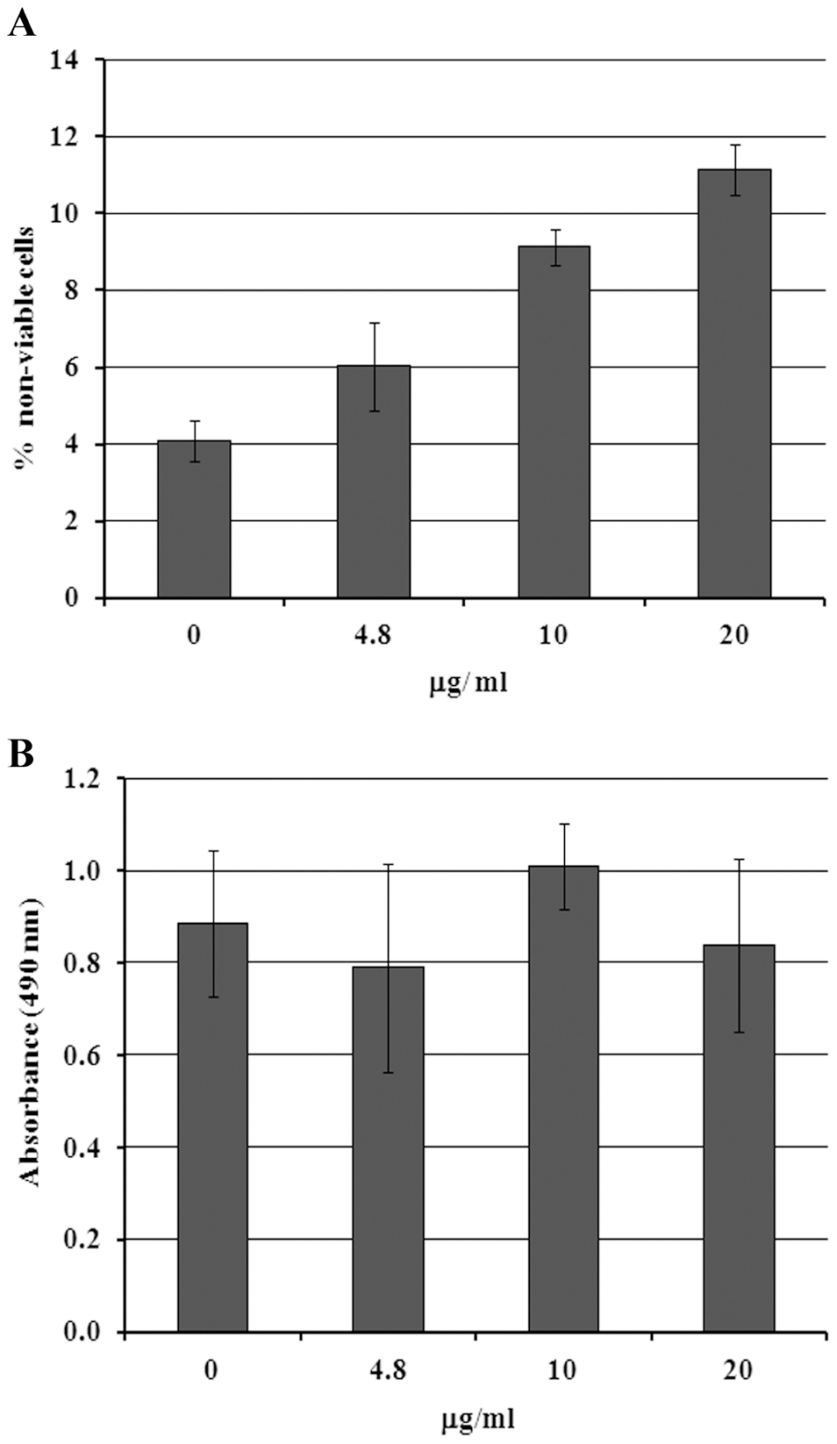
Cytotoxicity analyses of crovirin on LLC-MK_2_ cells (A) and peritoneal macrophages (B) by trypan blue cell viability and MTS assay, respectively. No tested concentrations induced significant lost of cell viability in either cell type. Error bars represent standard deviation of the mean of 3 independent experiments.

LLC-MK_2_ cells were treated with crovirin for 72 h and examined for viability using a trypan blue exclusion assay (**Fig. 3A**). None of the tested crovirin concentrations (4.8, 10 or 20 µg/ml) were capable of inducing significant loss of cell viability, even after 72 h of treatment. In addition, we tested the activity of crovirin against murine peritoneal macrophages to investigate its cytotoxicity towards primary host cells. Treated cells were examined using an MTS assay, and no significant toxicity (p≤0.05) was observed in any treatment conditions (**Fig. 3B**). Creatine kinase (CK) activity was measured before and two hours after *extensor digitorum longus* (EDL) muscle exposure to 10 µg/ml crovirin. We did not observed significant CK release from treated EDL muscles compared to control (saline) after 2 hours of incubation with crovirin, indicating that this protein did not generated appreciable myotoxicity at the concentration tested.

After establishing that crovirin had only minimal cytotoxic effects towards mammalian cells at concentrations of up to 20 µg/ml, we tested the anti-parasitic activity of purified crovirin against relevant developmental forms of three different species of pathogenic trypanosomatid parasites, namely *L. amazonensis*, *T. cruzi* and *T. brucei rhodesiense*.

**Figure 4:**
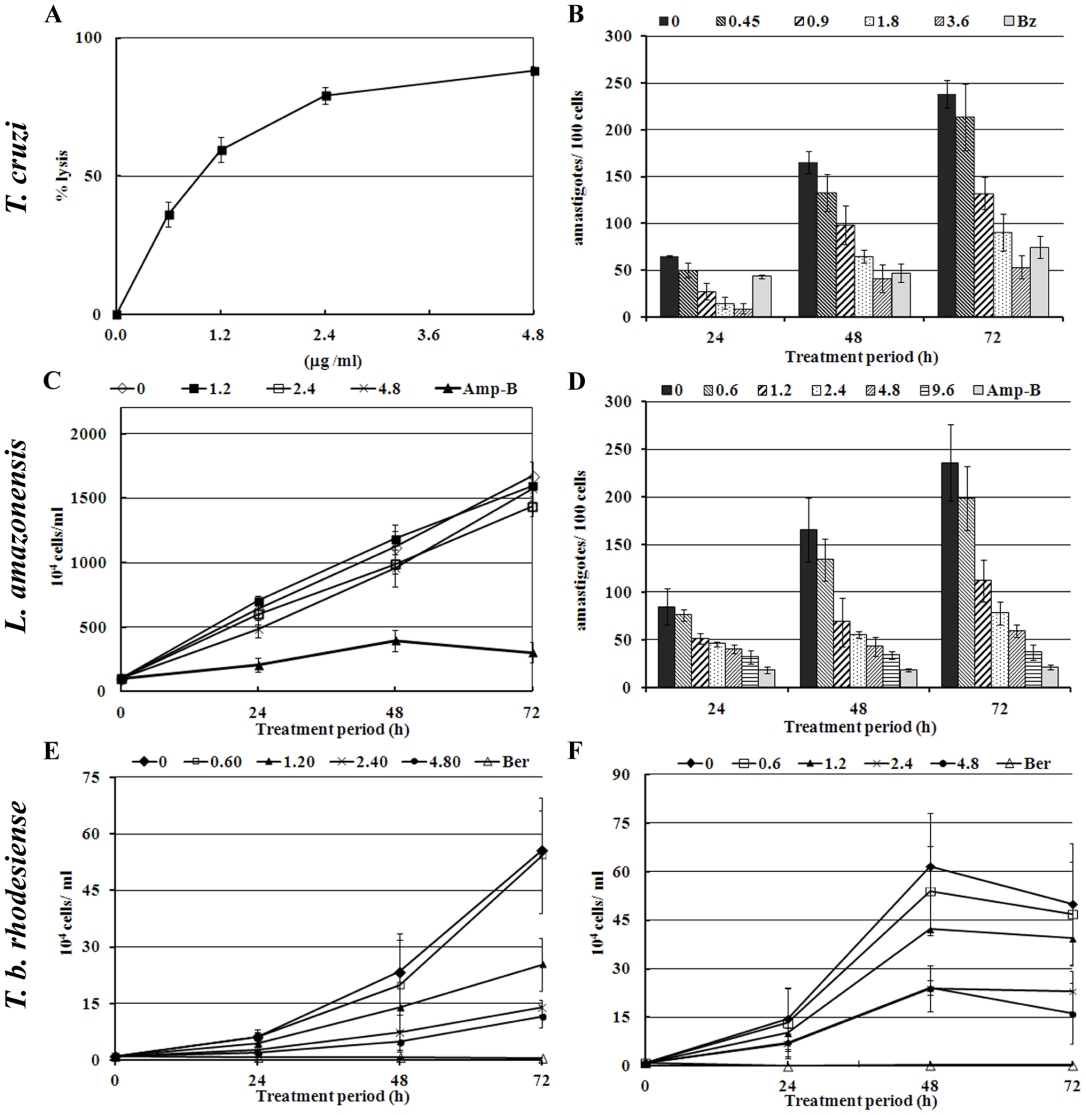
Crovirin effects on *T. cruzi* trypomastigotes (A) and intracellular amastigotes (B), *L. amazonensis* promastigotes (C) and intracellular amastigotes (D), and *T. brucei rhodesiense* PCF (procyclic form, in E) and BSF (bloodstream forms, in F). *T. cruzi* trypomastigotes were treated with crovirin in RPMI medium with 10% FCS for 24 h only, since they do not survive in growth medium for longer and do not divide. *T. brucei* BSF and PCF and *L. amazonensis* promastigotes were treated with crovirin in complete growth media, for up to 72 h. Amastigotes of *T. cruzi* and *Leishmania* were treated with crovirin as dividing intracellular forms (infecting peritoneal mouse macrophages). Error bars represent standard deviation the mean of 3 independent experiments.

We tested crovirin activity against the two infective *T. cruzi* forms, trypomastigotes and amastigotes. Trypomastigote forms do not multiply and do not remain viable after several days in culture media at 37°C. Therefore, the effect of crovirin towards *T. cruzi* trypomastigotes was evaluated as the ability of the protein to lyse cells after 24 h of treatment (**Fig. 4A**). The calculated LD_50_ of crovirin for trypomastigotes was 1.10±0.13 µg/ml (**Table 1**). This concentration displayed the second higher selectivity index (18.2) (**Table 1**) among all crovirin treatments.

**Table 1:** Crovirin activity towards medically important trypanosomes and *Leishmania*.

Species (clone or strain)	Developmental form treated	IC_50_ a or LD_50_ a (SI)^b^		
		24 h^c^	48 h^c^	72 h^c^
*T. cruzi* (CLBrener)	Trypomastigote	1.10±0.13 (18.2)	nd	nd
	Amastigote^d^	1.64±0.44 (12.2)	2.01±0.37 (9.9)	1.84±0.53 (10.9)
*L. amazonensis* (MHOM/BR/75/Josefa)	Promastigote	>4.8	>4.8	>4.8
	Amastigote^d^	2.38±0.62 (8.4)	1.05±0.17 (19.1)	1.21±0.89 (16.5)
*T. brucei rhodesiense* (427 - PCF; IL1852 - BSF)	PCF	2.18±0.50 (9.2)	1.70±0.13 (11.8)	1.13±0.31(17.7)
	BSF	2.18±0.39 (9.2)	2.24±0.56 (9.0)	2.06±0.12 (9.7)

a The IC_50_ and LD_50_ values are expressed in µg/ml.

b Selectivity Index (SI)^e^ was determined according to the ratio between the LC_50_^e^/IC_50_ or LD_50_ values. SI values, where LC_50_>20 µg/ml.

c Treatment period.

d parasite/100 cells.

nd: Not determined.

The treatment with 3.39 µg/ml Bz exhibited a 65.8% of parasites lysis at same conditions. *T. cruzi* amastigotes multiply in the intracellular environment. Crovirin inhibited the growth of amastigotes inside peritoneal macrophages in a dose-dependent manner (**Fig. 4B**), with an IC_50_ of 1.84±0.53 µg/ml when cells were treated with crovirin for 72 h (**Table 1**). Crovirin presented a discret superior trypanocidal activity against the intracellular forms as compared with Bz (**Fig. 4B**).

Crovirin activity was also tested against infective promastigote and amastigote forms of *L. amazonensis*, one of the species responsible for CL. None of the crovirin concentrations tested inhibited significantly the proliferation of *L. amazonensis* promastigotes in axenic media, unlike Amp-B treatment, which resulted in a reduction of a little over 80% in the number of parasites after 72 h of treatment. In contrast, crovirin inhibited the proliferation of intracellular amastigotes of *L. amazonensis* in a concentration-dependent manner (**Fig. 4C–D**). The effect of crovirin on amastigote proliferation was evident as early as 24 h after the start of treatment, and the IC_50_ for crovirin after 72 h of treatment was 1.21±0.89 µg/ml (**Table 1**). After 48 h incubation, the IC_50_ of 1.05 µg/ml also resulted in the highest selectivity index (19.1), being less toxic treatment to mammalian host cells. However, no tested concentration of crovirin had superior leishmanicidal activity against amastigotes forms as compared with Amp-B (**Fig. 4D**).

Both developmental forms of *T. brucei rhodesiense* tested here (PCF and BSF) were sensitive to crovirin treatment. A different profile of growth inhibition in the presence of crovirin was observed for PCF (**Fig. 4E**) and BSF (**Fig. 4F**) parasites, with IC_50_ values of 1.13±0.31 and 2.06±0.12 µg/ml, respectively, after 72 h of treatment. The 39.8 ng/ml Ber treatment resulted in a remarkable growth inhibition of both BCF and PCF than crovirin treatment (**Fig. 4E–F**).

## Discussion

There is an urgent need for the development of novel compounds for the treatment of trypanosomatid-borne diseases, currently treated with ‘dated’ chemotherapeutic agents with high toxicity and limited efficacy, partly due to the emergence of drug resistance. Animal venoms and toxins, including snake venoms, can provide compounds directly useful as drugs, or with potential as drug leads for the synthesis of novel therapeutic agents [22]. Previously, our group showed that Cvv crude venom displayed anti-parasitic activity against different *T. cruzi* developmental forms [46]. We have now extended this research with the purification of crovirin, a CRISP from Cvv venom with promising activity against key infective stages of the life cycle of *T. cruzi*, *T. brucei rhodesiense* and *L. amazonensis*. Furthermore, we show that crovirin has low toxicity towards host cells and mouse muscle, in agreement with the low or absent toxicity reported for most CRISPs proteins [35], [44]–[45].

CRISPs proteins are often given names that refer to the organism from which they were isolated. The first CRISP described in reptiles was isolated from the skin secretion of the lizard *Heloderma horridum*, and was named helodermin [56]. Examples of proteins isolated from snake venoms are patagonin, isolated from *Philodryas patagonensis* [44], latisemin, isolated from sea snake *Laticauda semifasciata*, tigrin isolated from *Rhabdophis tigrinus tigrinus* [41], and ablomin, isolated from *Gloydius blomhoffi* [41]. CRISPs sequences have also been identified in transcriptome analysis of venom glands [57]–[58] or are deposited at databanks but were not purified or studied. A partial CRISP sequence from *Crotalus viridis viridis* (GenBank gi:190195319) likely corresponding to central and C-terminal regions of crovirin was identified by transcriptome analysis of venom gland tissue. However, this is the first report on the purification and study of crovirin.

One of the most important findings of the present study was the activity of crovirin against the intracellular proliferation of trypanosomatids. Amastigotes are key developmental forms during the development and maintenance of infections by *Leishmania* and *T. cruzi*, representing the replicative intracellular stages of these protozoan parasites. Substantial inhibition of both *T. cruzi* and *L. amazonensis* intracellular amastigote proliferation was observed at crovirin concentrations significantly lower than those required to cause damage to host cells, including mouse EDL muscles. These results are particularly important because the currently available drugs to treat leishmaniasis and Chagas' disease are known to have lower anti-amastigote activity [1], [6].

The effects of crovirin over both the procyclic and the bloodstream form of *T. brucei rhodesiense* are also encouraging, suggesting that crovirin might be useful in the development of new anti-HAT chemotherapeutics. In conclusion, our results demonstrate that crovirin has promising trypanocidal and leishmanicidal effects, and represents a potential avenue for drug development against leishmaniasis, Chagas' disease and HAT, since its anti-parasitic effects are matched by low toxicity to host cells and muscles. Further studies are now required to extend our knowledge on the potential use of crovirin as an alternative compound to improve the effectiveness of treatment of trypanosomatid-borne neglected diseases.
